# Clinical and Hematological Manifestations of Systemic Lupus Erythematosus at Initial Presentation in a Tertiary Healthcare Center

**DOI:** 10.7759/cureus.75956

**Published:** 2024-12-18

**Authors:** Aruna Bathina, Dilip Chandra Chintada, Nikhil Kumar Reddy Yellu, Jami Vijayashree, Mohammed Khatija begum, Pooja Unnikrishnan

**Affiliations:** 1 Department of Dermatology, Venereology and Leprosy, Great Eastern Medical School & Hospital, Srikakulam, IND; 2 Department of Dermatology, Venereology and Leprosy, MediCiti Institute of Medical Sciences, Ghanpur, IND

**Keywords:** atypical presentation, autoimmune disease, clinical manifestations, early diagnosis, hematological abnormalities, systemic lupus erythematosus

## Abstract

Background

Systemic Lupus Erythematosus (SLE) is a multifaceted autoimmune disorder with diverse clinical presentations, among which hematological abnormalities often serve as early and critical indicators of disease. These manifestations, including anemia, leukopenia, lymphopenia, and thrombocytopenia, correlate with disease activity and provide essential diagnostic insights, particularly in resource-limited settings where access to advanced diagnostic tools may be constrained. This study emphasizes the significance of hematological findings that frequently appear at the initial presentation of SLE. They can guide early diagnosis and management, thereby improving patient outcomes.

Objective

This study aims to identify and analyze the clinical and hematological manifestations of SLE at initial presentation in a tertiary healthcare center. It focuses on hematological abnormalities as critical early indicators and highlights typical and atypical features that can aid in timely and accurate diagnosis.

Methods

A retrospective observational study was conducted at the dermatology department of the Great Eastern Medical School & Hospital. It included 53 patients diagnosed with SLE according to the Systemic Lupus International Collaborating Clinics (SLICC) criteria. Demographic data, clinical symptoms, hematological abnormalities, and immunological markers were recorded. The frequency and types of atypical manifestations were also noted.

Results

The study sample comprised 45 (85%) female patients and 8 (15%) male patients, with a mean age of 26 years. Common clinical manifestations included fever 49 (92%), joint pain 45 (85%), fatigue 43 (81%), oral ulcers 40 (75%), malar rash 40 (75%), and photosensitivity 44 (83%). Hematological abnormalities were prominent, with anemia observed in 52 (98%) of patients, leukopenia in 49 (92%), lymphopenia in 45 (85%), and thrombocytopenia in 20 (38%). Antinuclear antibody (ANA) test was positive in all cases, with elevated erythrocyte sedimentation rate (ESR) and CRP levels in newly-diagnosed patients. Atypical presentations included angioedema, toxic epidermal necrolysis (TEN)-like lesions, psoriasiform lesions, and pyrexia of unknown origin.

Conclusion

This study underscores the critical need to identify both typical and atypical clinical and hematological features of SLE, particularly at the initial presentation. Integrating comprehensive clinical evaluation with basic laboratory investigations can significantly enhance early detection, paving the way for timely and effective management to improve patient outcomes.

## Introduction

Systemic lupus erythematosus (SLE) is a multifaceted autoimmune disease characterized by a wide range of clinical and hematological manifestations, from mild skin involvement to severe multi-organ dysfunction. Its varied presentations make early diagnosis difficult, often demanding a high level of clinical suspicion. This study classifies SLE manifestations into typical symptoms, such as fever, joint pain, malar rash, and photosensitivity; and atypical presentations, including angioedema, toxic epidermal necrolysis (TEN)-like lesions, and psoriasiform eruptions [1,2]. The findings underscore the diagnostic complexities associated with atypical features and highlight the importance of heightened clinical awareness to identify the diverse spectrum of SLE presentations. The disease is marked by the production of autoantibodies targeting various cellular components, leading to inflammation and tissue damage in multiple systems, including the skin, joints, kidneys, cardiovascular system, and central nervous system [2]. Given its complex and variable presentation, SLE is often challenging to diagnose, with early detection relying on a high degree of clinical suspicion [3].

Hematological abnormalities are frequently observed in SLE and serve as the initial manifestation of the disease [4]. These include changes in the formed elements of blood, such as anemia, leukopenia, lymphopenia, and thrombocytopenia, as well as disturbances in clotting and fibrinolytic systems [5]. Anemia, in particular, is highly prevalent, occurring in more than 50% of SLE patients, with causes ranging from anemia of chronic disease to autoimmune hemolytic anemia [6]. Leukopenia and thrombocytopenia are also common, often correlating with disease activity [7]. The emphasis on hematological abnormalities in this study aligns with existing literature, which highlights their high prevalence and diagnostic significance in SLE [5-7].

Diagnosing SLE is challenging as no single test can confirm its presence. The American College of Rheumatology (ACR) and the Systemic Lupus International Collaborating Clinics (SLICC) criteria are the most widely accepted diagnostic guidelines, combining clinical features and laboratory findings [8]. However, the variability of symptoms often results in delayed or missed diagnoses, especially in atypical cases [9]. While the American College of Rheumatology criteria are highly specific, their lower sensitivity can leading early or mild cases being missed. On the other hand, the SLICC criteria improve sensitivity by including a wider range of clinical and immunological markers but may reduce specificity, increasing the risk of overclassification. These limitations are particularly evident in younger patients, whose clinical and immunological profiles differ from those of adults, and in early-stage SLE, where the symptoms may not yet meet classification thresholds [10].

This study addresses these gaps by emphasizing hematological abnormalities as early and accessible diagnostic indicators, particularly in resource-limited settings. It focuses on describing the clinical and hematological manifestations of SLE in a cohort of patients presenting to a tertiary healthcare center in South India. By exploring both common and atypical features at initial presentation, the study aims to deepen the understanding of SLE's diverse symptomatology and provide valuable insights to assist clinicians in early diagnosis. The emphasis on capturing a wide spectrum of manifestations, from typical symptoms like fever and malar rash to rarer presentations such as angioedema and TEN-like lesions, ensures a comprehensive analysis. This approach highlights the unique epidemiological and clinical context of SLE in the region, ultimately aiming to improve early recognition and management of this complex disease in tertiary care settings.

## Materials and methods

Study design

This retrospective observational study was conducted in the dermatology department of Great Eastern Medical School & Hospital, Srikakulam, over a period of six months, from April 2024 to September 2024. The study aimed to analyze the initial clinical and hematological manifestations in patients diagnosed with SLE according to the SLICC classification criteria.

As a retrospective study, this research involved reviewing and analyzing pre-existing data from patient medical records. The observational nature of the study focused on descriptive analysis of the prevalence and patterns of clinical and hematological symptoms in newly-diagnosed SLE cases, without intervention or follow-up assessments.

Study population

The study included 53 patients with newly-diagnosed SLE meeting the SLICC criteria during the six-month study period [10]. As a retrospective observational study, the sample size was determined by the availability of eligible cases, with patients diagnosed before April 2024 excluded to focus on initial disease presentation and minimize bias from prior treatment or progression. By examining both typical features, such as fever and malar rash, and atypical presentations like angioedema and TEN-like lesions, the study highlights the importance of recognizing SLE's diverse clinical manifestations. This approach aimed to enhance diagnostic accuracy and reduce delays in identifying atypical cases.

Demographic characteristics

The demographic profile of patients was carefully documented to analyze patterns related to the prevalence and presentation of SLE. This included recording key variables such as age, gender, and other relevant background characteristics. These details provided insights into how demographic factors influence the occurrence and initial manifestations of SLE, which are crucial for understanding the disease's epidemiology and tailoring patient care effectively.

Clinical manifestations

The study meticulously recorded specific clinical manifestations observed during the initial presentation of SLE.

Common Symptoms

Fever, joint pain, fatigue, oral ulcers, malar rash, photosensitivity, non-scarring alopecia, Raynaud’s phenomenon, and discoid rash, frequently associated with SLE [11].

Atypical Presentations

Rare manifestations included angioedema, TEN-like lesions, psoriasiform lesions, and pyrexia of unknown origin [12]. These atypical symptoms posed diagnostic challenges and necessitated heightened clinical suspicion for accurate identification of SLE.

Hematological findings

The hematological subtypes of anemia were classified based on clinical presentation, laboratory findings, and standard diagnostic criteria.

Anemia

Anemia of chronic disease was identified by low serum iron levels with normal or increased ferritin, autoimmune hemolytic anemia was confirmed by positive direct Coombs test, and microangiopathic hemolytic anemia was diagnosed through the presence of schistocytes on peripheral smear along with supporting clinical evidence [13].

Leukopenia

Leukopenia is defined as a condition in which the white blood cell (WBC) count falls below the normal reference range, indicating a reduction in the body’s ability to fight infections and maintain immune surveillance [14]. This abnormality is frequently observed in SLE and may reflect immune-mediated destruction of WBCs or bone marrow suppression caused by the disease process. Leukopenia is considered an important hematological marker for assessing disease activity in SLE, as it often correlates with flares and systemic inflammation.

Lymphopenia

Lymphopenia is characterized by a reduced number of lymphocytes in the blood, i.e., falling below the normal reference range [15]. Lymphocytes, a type of white blood cells, play a critical role in immune responses. In SLE, lymphopenia is a common finding and is attributed to autoantibody-mediated destruction, increased apoptosis, or impaired lymphocyte production. This abnormality can contribute to increased susceptibility to infections and may serve as an indicator of disease severity and systemic immune dysregulation in affected individuals.

Thrombocytopenia

Thrombocytopenia refers to a platelet count below the normal reference range, which can impair blood clotting and increase the risk of bleeding [13-15]. In the context of SLE, thrombocytopenia may result from immune-mediated destruction of platelets or decreased production in the bone marrow. It is a common hematological manifestation of SLE and is often associated with disease activity. Monitoring platelet levels is essential for managing SLE patients, as severe thrombocytopenia can lead to life-threatening bleeding complications.

Immunological markers

Immunological markers are vital for diagnosing SLE, assessing disease activity and severity, and guiding treatment strategies.

Antinuclear Antibody (ANA)

The presence of ANA was recorded in all patients, as it is one of the primary and most sensitive diagnostic markers for SLE [16]. ANAs are autoantibodies that target nuclear components within cells, and their detection is a hallmark of autoimmune diseases, particularly SLE. Although ANA lacks specificity-it can be positive in other autoimmune conditions-its presence, in conjunction with clinical features and other laboratory findings, is crucial for confirming an SLE diagnosis.

ANA testing was performed using the indirect immunofluorescence (IIF) method on HEp-2 cell substrates, which is considered the gold standard for ANA detection. Results were reported as titers with corresponding fluorescence patterns, including homogeneous, speckled, and nucleolar patterns [16]. These patterns provided crucial insights into the immunological profile of patients and were instrumental in meeting the SLICC classification criteria.

ESR and CRP

ESR and CRP levels were documented as key inflammatory markers, reflecting the systemic inflammatory activity in SLE [16]. An elevated ESR is commonly observed in SLE patients and is often indicative of chronic inflammation. However, CRP levels, while elevated in some cases, are typically less pronounced unless secondary infections or inflammatory conditions are present. The combined assessment of ESR and CRP helps differentiate between active disease states and other underlying conditions, making them valuable tools in monitoring disease activity and guiding treatment strategies.

Renal investigations

Urine routine and microscopic examinations were performed for all patients to evaluate renal involvement, focusing on proteinuria, hematuria, and cellular casts. Renal biopsy was conducted in patients with significant proteinuria (>500 mg/24 hours) or active urinary sediment indicative of lupus nephritis. Biopsy findings were classified according to the International Society of Nephrology/Renal Pathology Society (ISN/RPS) classification system, providing further insights into the spectrum of renal manifestations in the cohort.

Ethical approval

The study adhered to ethical standards to protect patient rights and confidentiality. Ethical approval was obtained from the institutional ethics committee (92/IEC/GEMS&H/2024 dated 13/4/2024) of Great Eastern Medical School & Hospital, Srikakulam. Informed consent was sought and signed by each patient before data extraction, ensuring participants' privacy and confidentiality throughout the research process.

Statistical analysis

Descriptive statistics were employed to analyze the data, focusing on frequency distribution and percentages for each variable. Clinical symptoms, hematological abnormalities, and atypical presentations were each evaluated for prevalence, allowing for a detailed profile of initial SLE manifestations. Results were compiled and presented in a tabular form for clarity, enhancing interpretation and allowing for comparisons of findings across different manifestations.

## Results

Demographic data

A total of 53 patients diagnosed with SL) were included in this study. The sample predominantly consisted of female patients with a mean age at presentation between 20 and 30 years, aligning with the higher incidence of SLE in young adult females (Table 1).

**Table 1:** Demographic data of the study population

Characteristic	Value
Total sample size	53
Females	45
Males	8
Mean age at presentation (years)	20-30

Clinical manifestations

The most frequent clinical symptoms at initial presentation were fever, joint pain, fatigue, and oral ulcers. Malar rash and photosensitivity were also common. Some less common symptoms included non-scarring alopecia, Raynaud’s phenomenon, discoid rash, and purpuric rash (Table 2).

**Table 2:** Frequency of clinical manifestations at presentation

Clinical manifestation	Frequency (n=53)	Percentage (%)
Fever	49	92%
Joint pains	45	85%
Fatigue	43	81%
Oral ulcers	40	75%
Malar rash	40	75%
Photosensitivity	44	83%
Non-scarring alopecia	35	66%
Raynaud’s phenomenon	4	8%
Discoid rash	12	23%
Purpuric rash	6	11%

Atypical clinical manifestations

We ensured the accuracy of identifying atypical features like TEN-like lesions and psoriasiform patterns through a thorough clinical evaluation by experienced dermatologists and review of patient records, including detailed descriptions and photographic evidence when available. Standard diagnostic criteria and differential diagnoses were applied to confirm these features, minimizing the risk of misclassification.

**Figure 1:**
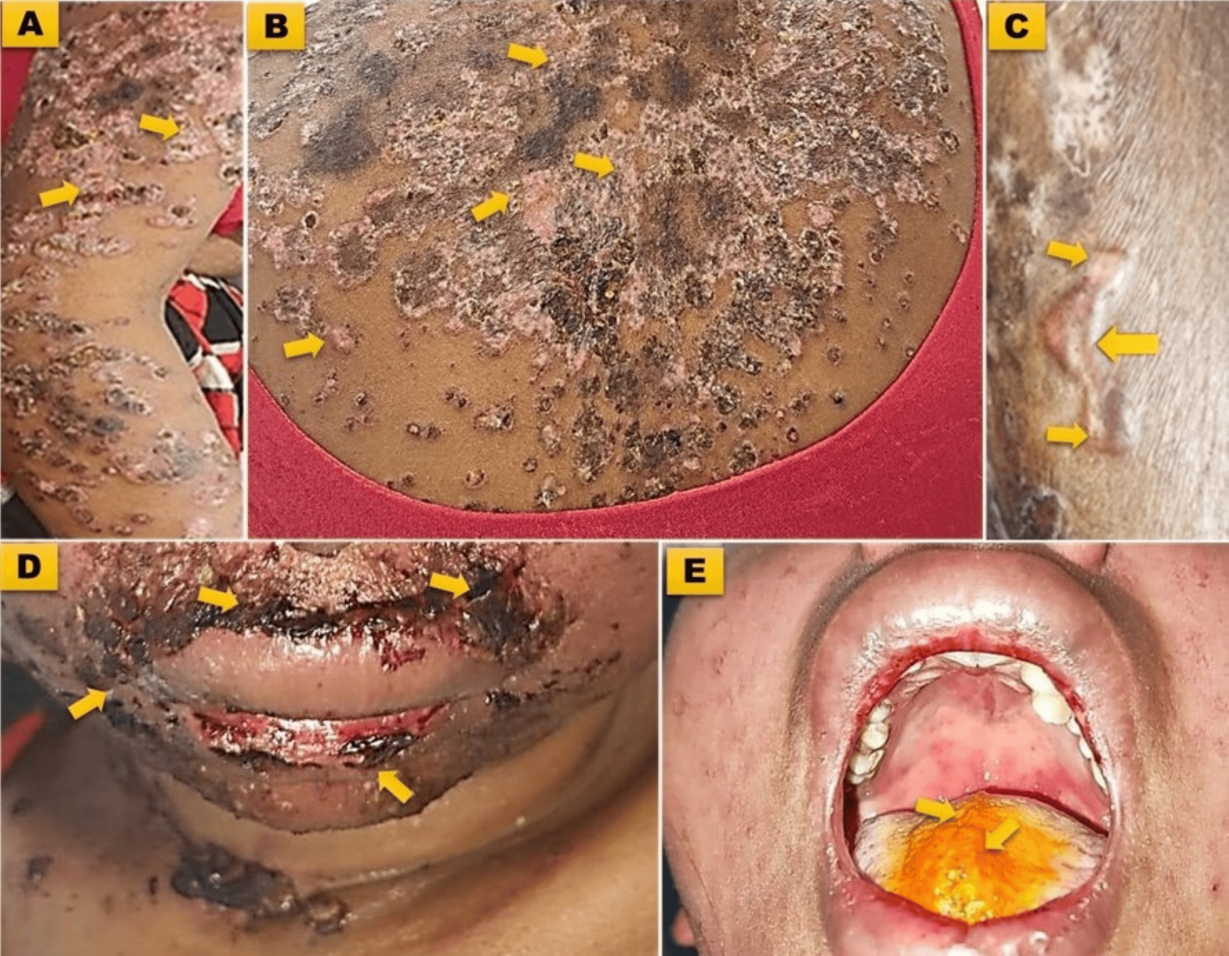
Clinical manifestations of systemic lupus erythematosus A) Psoriasiform lesions over the left limb; B) Psoriasiform lesions over the back; C) Bullous lupus erythematosus over the lower limb; D) Hemorrhagic crusted lesions over both the lips (angioedema); E) Ulceration of the hard palate.

A subset of patients presented with atypical manifestations, which contributed to diagnostic challenges. These included psoriasiform lesions (n=3; Figures 1A, 1B), bullous lesions (n=3; Figure 1C), a TEN-like presentation (n=2), angioedema (n=3; Figure 1D), and pyrexia of unknown origin (n=4; Table 3). Atypical features such as palatal ulceration (Figure 1E) highlight the diversity of SLE presentations, which can complicate early diagnosis.

**Table 3:** Atypical clinical manifestations of systemic lupus erythematosus TEN, Toxic epidermal necrolysis

Atypical manifestation	Frequency (n=53)	Percentage (%)
Angioedema	3	1.59
TEN-like presentation	2	1.06
Psoriasiform lesions	3	1.59
Pyrexia of unknown origin	4	2.12
Bullous lesions	3	1.59

Hematological manifestations

**Figure 2:**
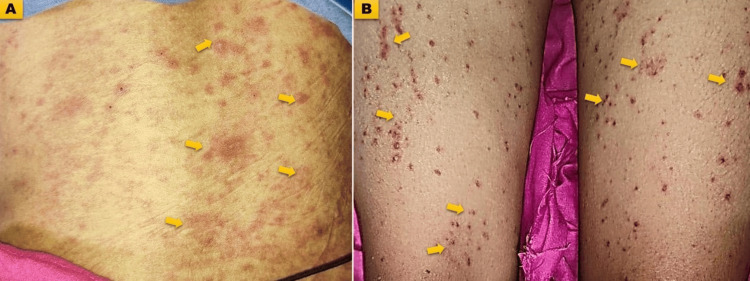
Clinical manifestations of systemic lupus erythematosus A) Hemorrhagic lesions over the abdomen (multiple purpuric); B) Hemorrhagic lesions over the lower limbs.

Hematological abnormalities were prominent among patients at initial presentation (Figures 1C, 2A, 2B) (Table 4).

**Table 4:** Hematological manifestations of systemic lupus erythematosus

Hematological abnormality	Frequency (n=53)	Percentage (%)
Anemia	52	98%
Leukopenia	49	92%
Lymphopenia	45	85%
Thrombocytopenia	20	38%

The high prevalence of anemia (98%) in this study underscores its role as a common and significant hematological manifestation of SLE, reflecting chronic inflammation, immune dysregulation, or direct autoimmune destruction of red blood cells. This finding aligns with the existing literature, which reports anemia as a frequent complication in SLE, though the prevalence in our cohort is notably higher than some studies. This variation may be attributed to the differences in study populations, disease severity, or the diagnostic criteria used. It emphasizes the importance of routine hematological screening for early detection and management of SLE patients.

Subtypes of Anemia

Anemia in these patients presented with varied etiologies. Most cases were attributed to anemia of chronic disease, which is associated with prolonged inflammation. Other subtypes included autoimmune hemolytic anemia, driven by immune-mediated red cell lysis, and microangiopathic hemolytic anemia, caused by vascular damage (Table 5).

**Table 5:** Subtypes of anemia observed in systemic lupus erythematosus

Type of anemia	Description
Anemia of chronic disease	Associated with prolonged inflammation
Autoimmune hemolytic anemia	Due to immune-mediated red cell lysis
Microangiopathic hemolytic anemia	Caused by damaged blood vessels

Immunological and inflammatory markers

All patients tested positive for ANAs, a common marker in SLE diagnosis. Additionally, elevated ESR and CRP levels were found in all newly-diagnosed cases, supporting the inflammatory nature of the disease at the onset (Table 6).

**Table 6:** Immunological and inflammatory markers in systemic lupus erythematosus ANA, Antinuclear antibodies; ESR, Erythrocyte sedimentation rate; CRP, C-reactive protein

Marker	Frequency (n=53)	Percentage (%)
ANA positive	53	100%
Elevated ESR and CRP	53	100%

Renal involvement

Renal involvement was observed in two patients who presented with lupus nephritis as an initial manifestation, characterized by elevated creatinine and blood urea levels. This finding aligns with the common involvement of renal complications in SLE and emphasizes the importance of early detection for improved outcomes (Table 7).

**Table 7:** Renal involvement in the initial presentation of systemic lupus erythematosus

Renal Manifestation	Frequency (n=53)	Description
Lupus nephritis	2	High creatinine and blood urea

## Discussion

SLE is a complex autoimmune disease marked by a range of clinical and hematological manifestations that vary widely among patients, making early diagnosis challenging. These diverse presentations often mimic other diseases, complicating the timely identification and management of SLE.

In a study conducted by AlOmair et al. (2023) [17], SLE was examined in a tertiary care setting and the multifaceted nature of the disease was highlighted through varied clinical manifestations and hematologic abnormalities that often complicate early diagnosis. Consistent with previous research by Fayyaz et al. (2015) [18] and Ahmmad et al. (2022) [19], this study confirmed that SLE predominantly affects young adult female patients, with an average age of onset between 20 and 30 years. The prevalence in female population underscores a potential link between SLE and hormonal or genetic factors, as suggested by Batool et al. (2016) [20]. Our findings are consistent with previous studies [17-20], showing a predominance of young female patients in the cohort, with male patients comprising a significantly smaller proportion. Anemia and malar rash were observed more frequently in the female patients, aligning with existing literature. Although detailed demographic correlations were not extensively analyzed in this study, future research is planned to investigate these associations in greater depth.

Joint pain, fatigue, malar rash, photosensitivity, and oral ulcers emerged as common symptoms in the initial presentation, supporting findings from earlier studies (Santacruz et al., 2022) [21]. These symptoms frequently serve as early indicators, aiding clinicians in identifying the disease at its onset. Dermatological features, such as non-scarring alopecia, discoid rash, and Raynaud's phenomenon, were also prominent, which aligns with Arathi et al. (2016) [22] and further emphasizes the importance of recognizing dermatologic and vascular symptoms as potential early signs of SLE, even in the absence of more typical presentations.

The hematologic profile of SLE patients often reveals significant abnormalities, as highlighted by AlOmair et al. (2023) [17]. Nearly all patients presented with anemia (98%), with additional high rates of leukopenia (92%) and lymphopenia (85%), findings corroborated by Fayyaz et al. (2015) [18] and Santacruz et al. (2022) [21]. Anemia, particularly in the form of chronic disease, autoimmune hemolytic anemia, and microangiopathic hemolytic anemia, provides valuable insights into SLE’s underlying pathogenic mechanisms, where inflammation and immune dysregulation play central roles. Leukopenia and thrombocytopenia, as noted by AlOmair et al. (2023) [17], serve as markers for disease activity, emphasizing the necessity of routine hematologic monitoring to guide treatment decisions.

Our data on hematological abnormalities, such as the high prevalence of anemia (98%), leukopenia (92%), and lymphopenia (85%), are consistent with findings from other studies, though the reported frequencies are slightly higher in our cohort. For instance, AlOmair et al. (2023) [17] and Fayyaz et al. (2015) [18] also highlighted anemia as the most common hematological abnormality in SLE. The slightly elevated rates in our study may reflect the differences in the study population, disease severity, or diagnostic methods, reinforcing the need for regular hematological evaluations in SLE patients for effective management.

Atypical clinical manifestations, such as angioedema, TEN-like presentations, and psoriasiform lesions, added complexity to the diagnostic process. Batool et al. (2016) [20] and Santacruz et al. (2022) [21] have similarly reported the diagnostic challenges posed by these atypical features, as they can mimic other conditions and lead to misdiagnoses [21]. For example, angioedema might be misinterpreted as an allergic reaction, while TEN-like lesions could mimic drug-induced skin conditions. Therefore, clinicians need to maintain a heightened level of suspicion for SLE, particularly in cases where atypical presentations obscure or mask standard diagnostic markers. The high prevalence of atypical features observed in this cohort may be linked to the thorough diagnostic approach adopted, which facilitated detailed identification and documentation of less common manifestations. Additionally, regional and demographic factors, such as genetic predisposition or environmental influences, could have contributed to these features.

Immunological markers such as ANA, elevated ESR, and CRP levels were identified as critical diagnostic tools in SLE, supporting findings by AlOmair et al. (2023) [17] and Fayyaz et al. (2015) [18]. CRP levels, typically associated with IL-6-mediated inflammation, have traditionally been considered less correlated with disease activity in SLE. In this study, CRP was elevated in all the newly-diagnosed patients, highlighting its potential utility as an early inflammatory marker in the initial recognition of SLE. While not a direct indicator of disease activity, its elevation alongside other inflammatory markers may aid in identifying early-stage SLE, particularly in cases with overlapping or atypical clinical features. ANA, although a highly sensitive indicator, lacks specificity and is positive in other autoimmune diseases. Elevated ESR and CRP levels reflect the inflammatory nature of SLE, aiding in differentiating it from other conditions, yet their interpretation should be contextualized with clinical and hematologic findings for a comprehensive diagnosis. As highlighted by Arathi et al. (2016) [22], reliance on ANA and related immunological markers requires a comprehensive approach, as not all SLE patients exhibit high ANA titers, emphasizing the importance of a multi-faceted diagnostic strategy for managing this complex disease. To address this, the study utilized ANA alongside the SLICC diagnostic criteria, which integrate clinical and additional immunological findings. This combined approach aimed to reduce diagnostic uncertainty and ensure accurate differentiation of SLE from the overlapping autoimmune disorders.

This study highlights the importance of a comprehensive approach to overcoming the challenges of diagnosing SLE, especially in cases with unusual symptoms. This includes careful clinical observation, detailed patient history, and consideration of both common and rare features, such as angioedema and TEN-like lesions, during diagnosis. Combining clinical findings with basic laboratory tests, like blood and immunological analyses, improves the accuracy of diagnosis and helps avoid delays or missed cases. In resource-limited settings, focusing on patient history, physical exams, and simple tests, such as blood profiling and inflammation markers, is essential. Early diagnosis and timely treatment of SLE are key to improving outcomes, preventing severe organ damage, and avoiding irreversible complications.

Limitations

This study’s retrospective design limits its ability to establish causal relationships and may introduce biases due to reliance on pre-existing medical records. As a single-center study with a relatively small sample size, the findings may lack generalizability and might not fully capture the diverse presentations of SLE in broader populations. The use of medical records may also have led to the omission of subtle clinical details, potentially affecting the comprehensiveness of data regarding the full clinical spectrum of SLE manifestations. Moreover, during the study period, some patients were non-cooperative or experienced discomfort during assessments, which may have limited the depth of data collection and contributed to variability in the recorded findings. These challenges underscore the inherent difficulties of conducting detailed and consistent research, particularly in resource-limited settings.

## Conclusions

This study underscores the diverse clinical and hematological manifestations of SLE at initial presentation, highlighting common symptoms such as fever, joint pain, fatigue, and hematologic abnormalities like anemia, leukopenia, and lymphopenia. The significant prevalence of atypical presentations, including angioedema and TEN-like lesions, illustrates the diagnostic complexity of SLE and the necessity for clinicians to adopt a vigilant, multi-faceted diagnostic approach. By emphasizing the integration of routine clinical evaluations, basic hematological profiling, and immunological markers, this study aligns its recommendations with resource-constrained settings, ensuring that early diagnosis and timely management of SLE remain achievable. Early intervention is critical to improving patient outcomes, particularly in regions with limited access to advanced diagnostic facilities.

## Appendices

**Table 8:** Spreadsheet summarizing the points of the modified SLICC criteria met by different patients SLICC, Systemic Lupus International Collaborating Clinics; ANA, antinuclear antibodies; Anti-dsDNA, anti-double stranded DNA; Anti-Sm, anti-Smith

Cases	Gender	Acute cutaneous lupus	Chronic cutaneous lupus	Oral ulcers	Non- scarring alopecia	Synovitis	Serositis	Renal abnormalities	Neurological abnormalities	Hemolytic anemia	Leukopenia or lymphopenia	Thrombocytopenia	ANA positivity	Anti- dsDNA	Anti-Sm	Anti-phospholipid antibodies	Low complement	Direct Coombs test
Case 1	Female	No	Yes	No	No	No	No	Yes	No	No	No	Yes	Yes	Yes	Yes	Yes	No	Yes
Case 2	Female	Yes	Yes	No	Yes	Yes	No	Yes	Yes	No	Yes	Yes	Yes	No	No	No	No	Yes
Case 3	Female	No	No	No	No	Yes	Yes	No	No	No	Yes	Yes	Yes	Yes	Yes	Yes	Yes	No
Case 4	Female	No	Yes	Yes	Yes	No	No	No	No	Yes	Yes	No	Yes	No	Yes	Yes	No	No
Case 5	Female	No	Yes	Yes	No	Yes	No	No	Yes	Yes	Yes	Yes	Yes	No	No	No	No	No
Case 6	Female	Yes	Yes	No	No	Yes	Yes	No	No	No	Yes	Yes	Yes	Yes	No	No	No	No
Case 7	Female	No	Yes	Yes	No	No	No	No	Yes	No	Yes	Yes	Yes	Yes	Yes	No	No	No
Case 8	Female	Yes	No	No	Yes	Yes	No	Yes	No	Yes	Yes	No	Yes	Yes	No	Yes	No	No
Case 9	Female	Yes	No	No	No	Yes	No	Yes	No	Yes	Yes	Yes	Yes	No	Yes	Yes	No	Yes
Case 10	Female	Yes	No	Yes	No	Yes	Yes	No	No	No	Yes	No	Yes	Yes	Yes	Yes	No	Yes
Case 11	Female	Yes	No	No	Yes	Yes	No	Yes	Yes	Yes	No	Yes	Yes	Yes	Yes	Yes	Yes	No
Case 12	Female	No	No	Yes	No	No	No	No	No	No	Yes	Yes	Yes	No	No	Yes	No	Yes
Case 13	Female	No	No	Yes	Yes	Yes	No	No	No	No	No	Yes	Yes	Yes	No	Yes	No	Yes
Case 14	Female	Yes	No	Yes	No	Yes	Yes	No	Yes	No	No	Yes	Yes	No	Yes	No	No	Yes
Case 15	Female	Yes	Yes	No	Yes	No	No	Yes	Yes	No	Yes	No	Yes	No	Yes	Yes	Yes	No
Case 16	Female	No	No	No	Yes	No	No	No	Yes	Yes	Yes	No	Yes	No	No	Yes	No	No
Case 17	Female	Yes	Yes	Yes	No	Yes	No	No	Yes	No	No	Yes	Yes	No	No	Yes	No	Yes
Case 18	Female	Yes	No	Yes	Yes	No	Yes	Yes	Yes	Yes	No	Yes	Yes	Yes	No	No	No	Yes
Case 19	Female	No	No	No	Yes	Yes	No	No	Yes	Yes	Yes	No	Yes	Yes	Yes	Yes	Yes	Yes
Case 20	Female	No	Yes	No	Yes	No	No	Yes	Yes	No	Yes	No	Yes	No	No	No	Yes	Yes
Case 21	Female	No	Yes	Yes	No	No	Yes	Yes	Yes	Yes	Yes	Yes	Yes	Yes	No	No	No	Yes
Case 22	Female	No	No	Yes	No	No	No	Yes	No	No	No	No	Yes	No	Yes	No	Yes	Yes
Case 23	Female	No	No	No	Yes	No	No	Yes	No	Yes	No	No	Yes	No	Yes	No	Yes	Yes
Case 24	Female	Yes	No	No	Yes	No	No	No	Yes	Yes	Yes	Yes	Yes	No	Yes	Yes	Yes	Yes
Case 25	Female	Yes	Yes	No	Yes	No	No	Yes	No	Yes	No	No	Yes	Yes	No	No	Yes	Yes
Case 26	Female	Yes	No	Yes	Yes	No	No	Yes	No	Yes	Yes	No	Yes	No	Yes	Yes	No	No
Case 27	Female	Yes	Yes	No	Yes	Yes	Yes	No	No	Yes	Yes	Yes	Yes	Yes	Yes	No	Yes	Yes
Case 28	Female	Yes	Yes	Yes	No	Yes	No	Yes	No	No	No	Yes	Yes	Yes	No	Yes	No	Yes
Case 29	Female	Yes	No	No	Yes	No	Yes	No	No	Yes	No	Yes	Yes	Yes	Yes	Yes	Yes	No
Case 30	Female	Yes	No	No	No	No	Yes	Yes	No	Yes	No	Yes	Yes	No	Yes	Yes	Yes	Yes
Case 31	Female	Yes	Yes	No	No	No	Yes	No	Yes	Yes	Yes	No	Yes	Yes	No	Yes	No	Yes
Case 32	Female	No	Yes	Yes	No	No	No	No	Yes	Yes	Yes	No	Yes	Yes	No	Yes	No	No
Case 33	Female	Yes	No	No	Yes	No	Yes	No	No	Yes	Yes	Yes	Yes	No	Yes	Yes	Yes	No
Case 34	Female	No	Yes	Yes	No	No	Yes	Yes	No	No	Yes	Yes	Yes	Yes	No	Yes	No	Yes
Case 35	Female	Yes	Yes	Yes	Yes	Yes	Yes	No	No	No	Yes	No	Yes	No	No	Yes	Yes	No
Case 36	Female	Yes	No	Yes	Yes	No	Yes	Yes	No	Yes	No	No	Yes	No	No	Yes	Yes	No
Case 37	Female	Yes	No	Yes	No	No	Yes	No	Yes	Yes	No	No	Yes	No	No	No	Yes	Yes
Case 38	Female	No	Yes	Yes	Yes	No	No	No	Yes	No	Yes	Yes	Yes	Yes	Yes	Yes	Yes	Yes
Case 39	Female	Yes	No	Yes	No	Yes	No	Yes	Yes	No	No	No	Yes	Yes	Yes	Yes	No	No
Case 40	Female	No	No	Yes	Yes	No	Yes	Yes	Yes	No	Yes	Yes	Yes	Yes	No	Yes	Yes	No
Case 41	Female	No	No	Yes	Yes	Yes	Yes	No	No	No	No	Yes	Yes	Yes	No	Yes	No	No
Case 42	Female	Yes	Yes	No	Yes	Yes	No	Yes	Yes	Yes	No	No	Yes	No	No	No	No	No
Case 43	Female	Yes	Yes	Yes	No	No	No	Yes	Yes	Yes	Yes	No	Yes	No	Yes	Yes	Yes	Yes
Case 44	Female	No	Yes	Yes	Yes	Yes	Yes	Yes	No	Yes	No	No	Yes	Yes	No	No	Yes	Yes
Case 45	Female	No	No	No	No	Yes	No	No	No	Yes	Yes	Yes	Yes	Yes	No	Yes	No	Yes
Case 46	Male	Yes	Yes	Yes	Yes	Yes	No	Yes	No	Yes	No	No	Yes	No	Yes	No	No	No
Case 47	Male	No	No	No	Yes	Yes	Yes	No	No	Yes	Yes	Yes	Yes	No	No	No	Yes	No
Case 48	Male	No	No	No	Yes	Yes	No	No	No	No	No	Yes	Yes	Yes	No	No	Yes	No
Case 49	Male	Yes	No	No	Yes	No	Yes	No	No	No	No	Yes	Yes	No	No	Yes	No	Yes
Case 50	Male	Yes	Yes	No	Yes	No	No	No	No	No	Yes	No	Yes	No	No	Yes	Yes	Yes
Case 51	Male	Yes	Yes	No	No	Yes	Yes	No	Yes	No	No	No	Yes	Yes	No	No	No	No
Case 52	Male	Yes	No	Yes	Yes	Yes	No	No	No	Yes	Yes	Yes	Yes	Yes	Yes	No	Yes	Yes
Case 53	Male	Yes	Yes	No	Yes	No	No	No	Yes	No	Yes	No	Yes	No	Yes	No	Yes	No
